# GASTRECTOMY IN OCTOGENARIANS WITH GASTRIC CANCER: IS IT FEASIBLE?

**DOI:** 10.1590/0102-672020200004e1552

**Published:** 2021-01-25

**Authors:** Francisco Diogo Almeida SILVA, Marina Alessandra PEREIRA, Marcus Fernando Kodama Pertille RAMOS, Ulysses RIBEIRO-JUNIOR, Bruno ZILBERSTEIN, Ivan CECCONELLO, Andre Roncon DIAS

**Affiliations:** 1 Faculty of Medical Sciences of Campina Grande, Medicine, Campina Grande, PB, Brazil; 2 Hospital de Clínicas, Faculty of Medicine, University of São Paulo, Cancer Institute, São Paulo, SP, Brazil

**Keywords:** Stomach neoplasms, Gastrectomy, Aged, 80 and over, Aged, Survival, Neoplasias gástricas, Gastrectomia, Idoso, Idoso de 80 anos ou mais, Sobrevida

## Abstract

***Background*:**

The octogenarian population is expanding worldwide and demand for gastrectomy due to gastric cancer in this population is expected to grow. However, the outcomes of surgery with curative intent in this age group are poorly reported and it is unclear what matters most to survival: age, clinical status, disease´s stage, or the extent of the surgery performed.

***Aim*:**

Evaluate the results of gastrectomy in octogenarians with gastric cancer and to verify the factors related to survival.

***Methods*:**

From prospective database, patients aged 80 years or older with histologically confirmed adenocarcinoma who had undergone gastrectomy with curative intent were selected. Factors related to postoperative complications and survival were studied.

***Results*:**

Fifty-one patients fulfilled the inclusion criteria. A total of 70.5% received subtotal gastrectomy and in 72.5% D1 lymphadenectomy was performed. Twenty-five (49%) had complications, in eleven major complications occurred (seven of these were clinical complications). Hospital length of stay was longer (8.5 vs. 17.8 days, p=0.002), and overall survival shorter (median of 1.4 vs. 20.5 months, p=0.009) for those with complications. D2 lymphadenectomy and the presence of postoperative complications were independent factors for worse overall survival.

***Conclusion*:**

Octogenarians undergoing gastrectomy with curative intent have high risk for postoperative clinical complications. D1 lymphadenectomy should be the standard of care in these patients.

## RESUMO

***Racional*:**

A população octogenária está expandindo mundialmente e é esperado que a demanda por gastrectomia devido a câncer gástrico nessa faixa também aumente. Entretanto, os resultados da operação curativa nessa população são pobremente reportados e não está claro o que mais importa no resultado: idade, status clínico, estágio da doença, ou a extensão da operação.

***Objetivos*:**

Avaliar os resultados cirúrgicos da gastrectomia em octogenários e verificar os fatores relacionados com a sobrevida.

***Métodos*:**

Através de revisão de banco de dados prospectivo, pacientes com 80 anos ou mais de idade e adenocarcinoma gástrico comprovado histologicamente e submetidos a gastrectomia com intuito curativo foram analisados. Fatores relacionados a complicações pós-operatórias e sobrevida foram estudadas.

Cinquenta e um pacientes preencheram os critérios de inclusão. A gastrectomia subtotal foi realizada em 70,5% dos casos e a linfadenectomia D1 em 72,5% dos pacientes. Complicações ocorreram em 25 pacientes (49%), sendo que em 11 elas foram graves (sete foram complicações clínicas). Pacientes com complicações tiveram maior duração da internação hospitalar (8,5 vs. 17,8 dias, p=0,002) e sobrevida global mais curta (mediana de 1,4 vs. 20,5 meses, p=0,009). Linfadenectomia D2 e a presença de complicações foram fatores independentes de pior sobrevida global.

***Conclusão*:**

Os octogenários submetidos à gastrectomia com intenção curativa apresentam alto risco de complicações clínicas no pós-operatório. A linfadenectomia D1 deve ser o padrão de atendimento nesses pacientes.

## INTRODUCTION

Gastric cancer is one of the leading causes of cancer-related mortality worldwide^10^ ^,^ ^21^. As most cases are diagnosed in the 7^th^ decade of life^4^ and life expectancy is increasing, demand for gastrectomy in the very older patients will rise^19^ ^,^ ^21^. Octogenarians are a particular group of interest. They are an expanding population, frequently frail or with comorbidities. Also, their complication rate is expected to be higher and survival shorter when compared to younger patients^8^ ^,^ ^13^ ^,^ ^20^. At this moment it is unclear what matters most to survival: age, preoperative clinical status, the disease`s stage, or the extent of the surgery performed. Is D2 lymphadenectomy adequate for octogenarians? All these topics remain poorly investigated.

This study aimed to evaluate the results of gastrectomy in octogenarians with gastric cancer and to verify the factors related to low survival.

## METHODS

This study was approved by the hospital ethics committee and is registered online (www.plataformabrasil.com; CAAE: 30308620.1.0000.0068).

### Patient population and study design

All patients who underwent any surgical procedure for gastric cancer between 2009 and 2019 were retrospectively evaluated. Data were obtained from a prospective medical database. Eligible patients were those aged 80 years or older, with histologically confirmed adenocarcinoma and submitted to gastrectomy with curative intent. Exclusion criteria included palliative surgery and procedures performed in urgency/emergency setting.

Comorbidities were evaluated by the Charlson comorbidity index and surgical complications were graded according to Clavien-Dindo (>2 was considered as major complication)^6^. Deaths until 30 days after the gastrectomy or during the postoperative stay were considered as surgical mortality.

The surgical procedure was performed as recommended by the Japanese Gastric Cancer Association^7^.Tumor was staged according to the 8^th^ TNM edition^1^.

Patients were divided into two groups: with and without postoperative complications (POC). Complications were classified as clinical or surgical (those directly related to the procedure).

### Statistical analysis

Nominal data are presented in frequencies with percentages and numerical data in means with standard deviation. Continuous and categorical variables were analyzed by t-test and squared-chi test, respectively. A receiver operating characteristic (ROC) curve was used to determine the better cutoff value for tumor size that correlated with death. The area under the ROC curve (AUC) was employed as a measure of accuracy. Survival was calculated from the date of surgery until the event (death/relapse) or last follow-up and estimated using the Kaplan-Meier method; the log-rank test was used to evaluate the difference between the curves. Overall survival (OS) was calculated until death and disease-free survival until the date of disease recurrence. Multivariate Cox proportional hazard analysis was performed to analyze the prognostic factors related to survival. Hazard ratio and 95% confidence interval were calculated as a measurement of association. All p values were reported as two-tailed and a p-value of 0.05 or less was considered statistically significant. Analyses were performed using the SPSS program (Version 20; SPSS, Chicago, IL, USA).

## RESULTS

In the period considered, from 1,156 patients operated, 91 were octogenarians (7.8%) and 51 fulfilled the inclusion criteria. Most patients were males (72.5%) and the mean age was 84 years old (range 80-94). Subtotal gastrectomy was performed in 70.5% of the cases and 72.5% had D1 lymphadenectomy. The mean number of lymph nodes retrieved was 35. The mean tumor size was 4.9 cm (±2.5). The ROC curve determined the cut-off of 4.9 cm for lesion size associated with death (AUC=70.7%, 95%CI 0.56 - 0.86, p=0.012).

POC occurred in 24 (47%) patients, eleven (21.5% of the total) had major ones. Clinical POC were responsible for seven of the major complications and four (out of six) postoperative deaths.

Clinical and pathological characteristics of those with and without POC are presented in Table 1. Age, gender, Charlson index, ASA, and TNM stage were similar between groups; lymphatic and venous invasion were more frequent in the POC group. Hospital length of stay was higher in the POC group (8.5 vs. 17.8 days, p=0.002).

**TABLE 1:** Clinicopathological characteristics of gastric cancer octogenarians according to the presence or absence of postoperative complications (POC)

		non-POC	POC	
Variables		n= 27	n= 24	p
Gender				0.104
	Female	10 (37)	4 (16.7)	
	Male	17 (63)	20 (83.3)	
Age (years)				0.843
	Mean (SD)	83.9 (2.7)	84.1 (3.8)	
BMI (Kg/cm²)				0.670
	Mean (SD)	23.7 (3.8)	23.3 (3.5)	
Hemoglobin				0.063
	Mean (SD)	12.1 (2.0)	10.8 (2.1)	
Neutrophil lymphocyte ratio (NLR)				0.715
	Mean (SD)	3.11 (3.17)	2.78 (1.99)	
Charlson-Deyo Comorbidity Index (CCI)				0.842
	0	15.6 (55.6)	14 (58.3)	
	>1	12 (44.4)	10 (41.7)	
American Society of Anesthesiologists (ASA)				0.593
	II	18 (66.7)	14 (58.3)	
	III	9 (33.3)	10 (41.7)	
Type of surgery				0.232
	Subtotal	21 (77.8)	15 (62.5)	
	Total	6 (22.2)	9 (37.5)	
Type of lymphadenectomy				0.318
	D1	18 (66.7)	19 (79.2)	
	D2	9 (33.3)	5 (20.8)	
Tumor site				0.267
	Lower	20 (74.1)	14 (58.3)	
	Middle	6 (22.2)	6 (25)	
	Upper	1 (3.7)	1 (4.2)	
	Stump	0 (0)	3 (12.5)	
Tumor size				0.781
	Smaller (<4.9cm)	14 (51.9)	11 (45.8)	
	Larger	12 (48.1)	13 (54.2)	
Lauren type				0.570
	Intestinal	21 (77.8)	17 (70.8)	
	Diffuse/mixed	6 (22.2)	7 (29.2)	
Histological grade				0.552
	Well / moderately differentiated	19 (70.4)	15 (62.5)	
	Poorly differentiated	8 (29.6)	9 (37.5)	
Lymphatic invasion				0.016
	No	17 (63)	7 (29.2)	
	Yes	10 (37)	17 (70.8)	
Venous invasion				0.038
	No	21 (77.8)	12 (50)	
	Yes	6 (22.2)	12 (50)	
Perineural Invasion				0.507
	No	16 (59.3)	12 (50)	
	Yes	11 (40.7)	12 (50)	
N° LNs				0.947
	Mean (SD)	34.9 (17.6)	35.3 (20.1)	
pT				0.388
	T1/T2	11 (40.7)	7 (29.2)	
	T3/T4	16 (59.3)	17 (70.8)	
pN				0.100
	N0	14 (51.9)	7 (29.2)	
	N+	13 (48.1)	17 (79.8)	
pTNM				0.645
	I	9 (33.3)	7 (29.2)	
	II	9 (33.3)	6 (25)	
	III	9 (33.3)	11 (45.8)	
Postoperative complication (POC)				na
	No POC	27 (0)	0 (0)	
	Minor POC	0 (0)	13 (54.2)	
	Major POC	0 (0)	11 (45.8)	
Chemotherapy				1.0
	No	25 (92.6)	22 (91.7)	
	Yes	2 (7.4)	2 (8.3)	

SD=standard deviation; p-values in bold are statistically significant

### Survival outcomes

**FIGURE 1:**
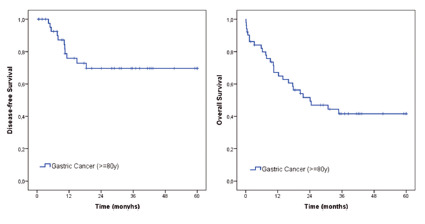
Overall survival and disease-free survival for gastric cancer octogenarians

In a median follow-up of eighteen months, 27 (53%) patients died, and eleven (21.5%) had disease recurrence. Mortality in 30 and 90 days were 9.8% (n=5) and 15.7% (n=8). The median OS for all patients was 24.1 months. Disease-free survival and OS curves are presented in Figure 1.

**FIGURE 2:**
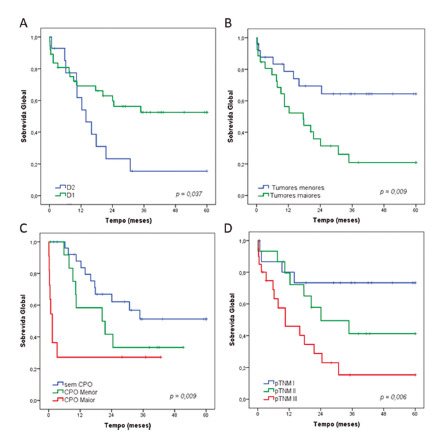
Overall survival for gastric cancer octogenarians according to: A) type of lymphadenectomy performed (D1 vs. D2); B) tumor size (< vs. ≥ 4.9 cm); C) Postoperative complications (absent vs. minor vs. major); D) TNM stage (I vs. II vs. III).

Regarding the type of lymphadenectomy, patients who underwent D1 y had better OS rates compared to D2 (p=0.037). The median OS for D2 was 13.9 months (median not reached for D1, Figure 2A).

Considering the tumor size, patients with larger lesions (≥ 4.9 cm) had worse OS (median of 17.2 months - median not reached for smaller lesions, p=0.015, Figure 2B).

Survival was different according to the occurrence of surgical complications (p=0.009). The median OS for patients with major-POC and minor-POC was 1.4 and 20.5 months, respectively (Figure 2C). Concerning the non-POC group as reference, patients with minor-POC had lower survival (p=0.120), followed by those with major-POC (p=0.003).

When stratified by pTNM, the OS was significantly different between stages I, II, and III (p=0.006). The median OS for stages II and III were 24.1 and 10.5 months, respectively (Figure 2D). 

At multivariate analysis, D2 lymphadenectomy, and the presence of POC were independent factors for worse OS (Table 2).

**TABLE 2:** Univariate and multivariate analysis for overall survival

Overall survival	Univariate analysis			Multivariate analysis		
Variables	HR	95% CI	p	HR	95% CI	p
Male (vs. female)	1.05	0.45 -2.49	0.906	-	-	-
ASA III (vs. ASA II)	1.44	0.67 - 3.11	0.354	-	-	-
Charlson >1 (vs. Charlson 0)	0.79	0.36 - 1.73	0.560	-	-	-
Total gastrectomy (vs. subtotal)	0.89	0.39 - 2.04	0.785	-	-	-
D2 (vs. D1)	2.25	1.03 - 4.91	0.042	3.53	1.41 - 8.83	0.007
Large tumor (≥ 4.9 cm) (vs smaller)	2.70	1.18 - 6.20	0.019	1.96	0.74 - 5.23	0.178
pN+ (vs. pN0)	1.82	0.81 - 4.06	0.144	-	-	-
pT3/T4 (vs T1/T2)	3.98	1.37 - 11.52	0.011	2.29	0.66 - 7.89	0.191
Major POC (vs. non/minor POC)	3.17	1.37 - 7.31	0.007	1.19	1.93 - 13.95	0.001

HR=hazard ratio; CI=confidence interval; ASA=American Society of Anesthesiologists; POC=postoperative complication; p-values in bold are statistically significant.

## DISCUSSION

Gastrectomy outcomes in octogenarians are a debatable matter with data coming from the small and unicentric retrospective series^21^. In our cohort, gastric cancer octogenarians undergoing surgery with curative intent were usually submitted to subtotal gastrectomy and received D1 lymphadenectomy. Complications were frequent (47%) a higher when compared to series with younger patients^16^ ^,^ ^17^. Major complications occurred in 21.5% of the patients and were usually due to clinical ones, which is similar to what other authors observed^2^ ^,^ ^11^ ^,^ ^12^ ^,^ ^14^. Patients with POC had longer hospital length of stay and shorter OS. Major complications were a significant predictor of poor survival. 

Patients with lesions ≥4.9 cm also had worse OS, size has been implicated as a predictor of survival by other authors as well^9^ ^,^ ^18^ ^,^ ^22^. D2 lymphadenectomy was an independent risk factor for shorter survival with a hazard ratio of 3.53. Although D2 is indicated for advanced gastric cancer^23^, it also carries an increased risk for complications, and since octogenarians are already frail, with comorbidities and have a short life expectancy, a quicker and less oncological procedure (D1) is better^8^ ^,^ ^21^. In fact, D1 in frail patients is already very morbid^12^. Additionally, in our cohort, the presence of lymph node metastasis was not a factor associated with survival, which reinforces that restricted lymphadenectomy is appropriate in these patients. In our opinion, omentectomy may also be neglected in these patients^3^.

Clinical parameters expected to impair survival (ASA, Charlson index) were not significant at univariate analysis, probably because this is a selected cohort of patients. Octogenarians with unfavorable conditions were not indicated for surgery with curative intent. Additionally, total gastrectomy was not associated with worst outcomes (when compared to subtotal) and it may be due to a small number of cases included, and there is again selection bias since distal and less morbid surgery was preferred for octogenarians.

It is worth mentioning that the number of lymph nodes retrieved was high even for D1. We credit this to the use of Carnoy´s solution as a fixative and not contamination of D2 nodal stations in D1 patients^5^_._

The present study has the limitations of its retrospective nature. On the other hand, it presents a population considered fit for curative surgery, preventing bias in the survival analysis.

## CONCLUSION

Octogenarians undergoing gastrectomy with curative intent have a high risk of postoperative clinical complications. D2 lymphadenectomy and the occurrence of major complications were independent risk factors associated with worse survival. D1 lymphadenectomy should be the standard in these patients.
